# Correlation of VAP diagnosis with parameters of critically ill patients in a general ICU

**DOI:** 10.1186/cc10634

**Published:** 2012-03-20

**Authors:** DK Matthaiou, A Ioannidis, G Gounti, D Lathyris, A Vathis, S Vasiliagkou, K Kontopoulou, K Mandraveli, E Antoniadou

**Affiliations:** 1 'G. Gennimatas' General Hospital, Thessaloniki, Greece

## Introduction

We aimed to describe various parameters of critically ill patients who developed VAP and correlate them with its outcome.

## Methods

Twenty-three VAP cases out of 338 ICU patients were studied retrospectively. Data regarding age, sex, etiology, scores (APACHE II, SOFA, CPIS), CRP, miniBAL cultures, comorbidities, antibiotic exposure, duration of mechanical ventilation, length of ICU and total stay, VAP and patient outcome were recorded. Chi-square and Mann-Whitney U tests were used for statistical analyses.

## Results

VAP incidence was 23/338 (6.8%). Fourteen of 23(60.9%) were males, and 9/23(39.1%) were surgical patients. Their age was 63.5 ± 16.6 years. APACHE II was 20.5 ± 6.7, initial SOFA was 8.8 ± 3.7, SOFA at VAP was 9.4 ± 3.1, CPIS 2 days before VAP was 4.6 ± 2, CPIS the day before VAP was 6 ± 1.2, and CPIS at VAP was 7.6 ± 1.3. Length of stay was 25.5 ± 13.1 days, ICU stay was 24.8 ± 13.4 days, and duration of mechanical ventilation was 22.5 ± 12.1 days. Previous antibiotic exposure included: linezolid 10/23 (43.5%), vancomycin 2/23 (8.7%), antipseudomonadic penicillins 14/23 (60.9%), β-lactams ± β-lactamase inhibitor 7/23 (30.4%), quinolones 14/23 (60.9%), aminoglycosides 6/23 (26.1%), antifungals 4/23 (17.4%), carbapenems 1/23 (4.3%), tigecycline 3/23 (13%), and colistin 8/23 (34.8%). Antibiotic therapy after the positive miniBAL was modified according to antibiograms. The isolated microorganisms in miniBAL were *A. baumannii *10/23 (43.5%), *P. aeruginosa *5/23 (21.7%), *K. pneumoniae *4/23 (17.4%), *Candida *spp. 2/23 (8.7%), and other 4/23 (17.4%); one infection was polymicrobial. In 20/23 cases (87%) VAP was of late onset (>4 days) (9.7 ± 6.8 days). VAP was improved in 17/23 cases (73.9%), but 15/23 patients (65.2%) died. High overall mortality may be attributed to grave condition. Most patients were admitted to the ICU hours after they were admitted to the hospital. Increased SOFA scores during admission (12 ± 2 vs. 7.7 ± 3.4, *P *= 0.009) and on the day of VAP diagnosis (11.5 ± 2.1 vs. 8.6 ± 3, *P *= 0.016) were associated with VAP deterioration. Increased CPIS on the last 2 days before VAP was also associated with worse VAP outcomes (6.2 ± 1.7 vs. 4.1 ± 1.8 and 6.8 ± 1.2 vs. 5.7 ± 1.1, *P *= 0.03 and *P *= 0.03, respectively).

## Conclusion

Our findings support the prognostic value of SOFA score. CPIS values of 6, although not diagnostic, may need increased alertness on behalf of the clinician.
